# Socioeconomic and programmatic determinants of renewal of membership in a voluntary micro health insurance scheme: evidence from Chakaria, Bangladesh

**DOI:** 10.1080/16549716.2017.1287398

**Published:** 2017-05-04

**Authors:** Mohammad Iqbal, Asiful Haidar Chowdhury, Shehrin Shaila Mahmood, Mohammad Nahid Mia, S. M. A. Hanifi, Abbas Bhuiya

**Affiliations:** ^a^ Health Systems and Population Studies Division, icddr,b, Dhaka, Bangladesh; ^b^ Partners in Population and Development, Dhaka, Bangladesh

**Keywords:** Micro health insurance, enrolment, membership renewal, determinants, benefit, service utilization

## Abstract

**Background:** Out-of-pocket (OOP) healthcare expenditure is a major obstacle for achieving universal health coverage in low-income countries including Bangladesh. Sixty-three percent of the USD 27 annual per-capita healthcare expenditure in Bangladesh comes from individuals’ pockets. Although health insurance is a financial tool for reducing OOP, use of such tools in Bangladesh has been limited to some small-scale voluntary micro health insurance (MHI) schemes run by non-governmental organizations (NGO). The MHI, however, can orient people on health insurance concept and provide learning for product development, implementation, barriers to enrolment, membership renewal, and other operational challenges and solutions. Keeping this in mind, icddr,b in 2012 initiated a pilot MHI, *Amader Shasthya*, in Chakaria, Bangladesh. This paper explores the determinants of membership renewal in this scheme, which is a perpetual challenge for MHI.

**Objective:** Identify socioeconomic and programmatic determinants and their effects on membership renewal in a voluntary MHI scheme.

**Methods:** Data came from the online management information system of the scheme and Health and Demographic Surveillance System of Chakaria, covering the period February 2012–May 2015. Association between renewal and independent variables was examined using cross-tabular and logistic regression analyses.

**Results:** Nearly 20% of households in the catchment area ever enroled in the scheme, and 38% renewed membership over the initial 3 years of operation. Frequency of consultation with healthcare providers, benefits received, proximity of member’s residence to health facility, socioeconomic status, educational level, and age of the household head showed significant positive association with renewal of membership.

**Conclusions:** Villagers’ enrolment in the scheme indicated that even in poor economic and literacy conditions people can be motivated to enrol in insurance schemes. Degree of service utilization and benefits received can greatly enhance the probability of membership renewal, which can be ensured with good quality of services and ease of access.

## Background

High out-of-pocket (OOP) healthcare expenditure is a major hurdle in achieving universal health coverage (UHC), an explicit target under the recently announced sustainable development goals (SDG) [1]. Bangladesh, along with many other developing countries, faces the challenge of extremely high OOP healthcare expenditure. Of the annual per-capita healthcare expenditure of USD 27 in Bangladesh, 63% (USD 17) [2] comes from direct payment by individuals, which is one of the highest proportions in Asia. Nearly 100 million people worldwide [3] and 6.4 million people in Bangladesh [4] slide below the poverty line every year in meeting healthcare costs. Further, high OOP healthcare expenditure can delay and, at times, deter access to much-needed healthcare. In a pluralistic health service environment, the public healthcare service of Bangladesh is not showing any sign of reducing OOP healthcare expenditure. Even though by constitution the public healthcare services are to be provided free of charge [5], supply shortages, an inadequate mix of healthcare providers, absenteeism among healthcare providers, and under-the table payment add to the OOP expenses. With Bangladesh being a signatory to UHC under the SDG, it is thus important to find ways to reduce OOP healthcare expenditure. Although health insurance is considered one of the health financing tools that can potentially reduce OOP expenditure, it is still in a nascent stage in terms of its scope and coverage in Bangladesh. The very concept of insuring against health-related risk is unknown to common people.

Micro health insurance (MHI), a customized voluntary health insurance scheme, has been used for serving people in other developing countries [6–10] and Bangladesh can use it to test the acceptability of the insurance mechanism as well as understand people’s behaviour in relation to the concepts of insurance, enrolment, and renewal [11]. The learning from MHI schemes can, thus, feed into implementation of large-scale health insurance schemes throughout the country.

It is against this background that in 2012 icddr,b, an international health research organization based in Bangladesh, initiated a pilot MHI scheme in Chakaria, a remote rural area. The primary aim of the initiative was to provide a useful scheme for Bangladesh to pool money from the villagers by using the concept of risk protection as an incentive for prepayment. The scheme is named *Amader Shasthya* (AS), meaning ‘Our Health’, to imply community ownership and solidarity. The scheme aimed to learn about community responses to the concept of health insurance in general and MHI in particular [12]. Nevertheless, one of the inherent challenges in running a voluntary MHI has been the low uptake and renewal rate of membership. High enrolment as well as membership renewal is important both for ensuring benefit to the people and for increasing the pool and thereby the financial viability of any MHI scheme [13]. Although AS succeeded in attracting a good number of members in a limited time period, it is struggling to keep the renewal rate at a replacement level. This paper, thus, attempts to identify the socioeconomic and programmatic determinants of renewal, which can be helpful in improving the programme to achieve enhanced renewal rates in low-resource settings.

## Methods

### Study area

The study area, Chakaria, is a sub-district located in the southeast coast of Bangladesh. The sub-district, under the Cox’s Bazar district of Bangladesh, has an estimated population of 511,861 [14]. icddr,b has been maintaining a Health and Demographic Surveillance System (HDSS) at the Chakaria site since 1999, and is a member of the INDEPTH network [15]. The area is rural in nature, with farming being the major means of livelihood. The literacy rate is 47.6% [16]. The health services in the area include those provided by both the public and private sectors. In the public sector, there is a 50-bed hospital at the sub-district headquarters, and there are 15 Union Health and Family Welfare Centres (UHFWC)/Family Welfare Centres (FWC) at the union level, and 40 Community Clinics (CC) at the village level. The CC are the outdoor primary healthcare centres of the government, each covering around 6000 people, and the UHFWC are the next higher-level facilities for outdoor primary care, each covering around 15,000 people. Both of these facilities are run by government-funded para-professionals. In addition to these, there are four village health posts (VHP) for outdoor primary care; these are run by icddr,b-appointed paramedics and physicians. Members of the MHI receive services from these paramedics five days a week and from the physicians once a week. The private-sector services are provided by five hospitals. Among these, Zamzam Hospital has the highest capacity with 100 beds, and each of the rest has a 50-bed capacity. Members of the inpatient package of the AS insurance scheme receive services from Zamzam Hospital.

**Figure 1.**
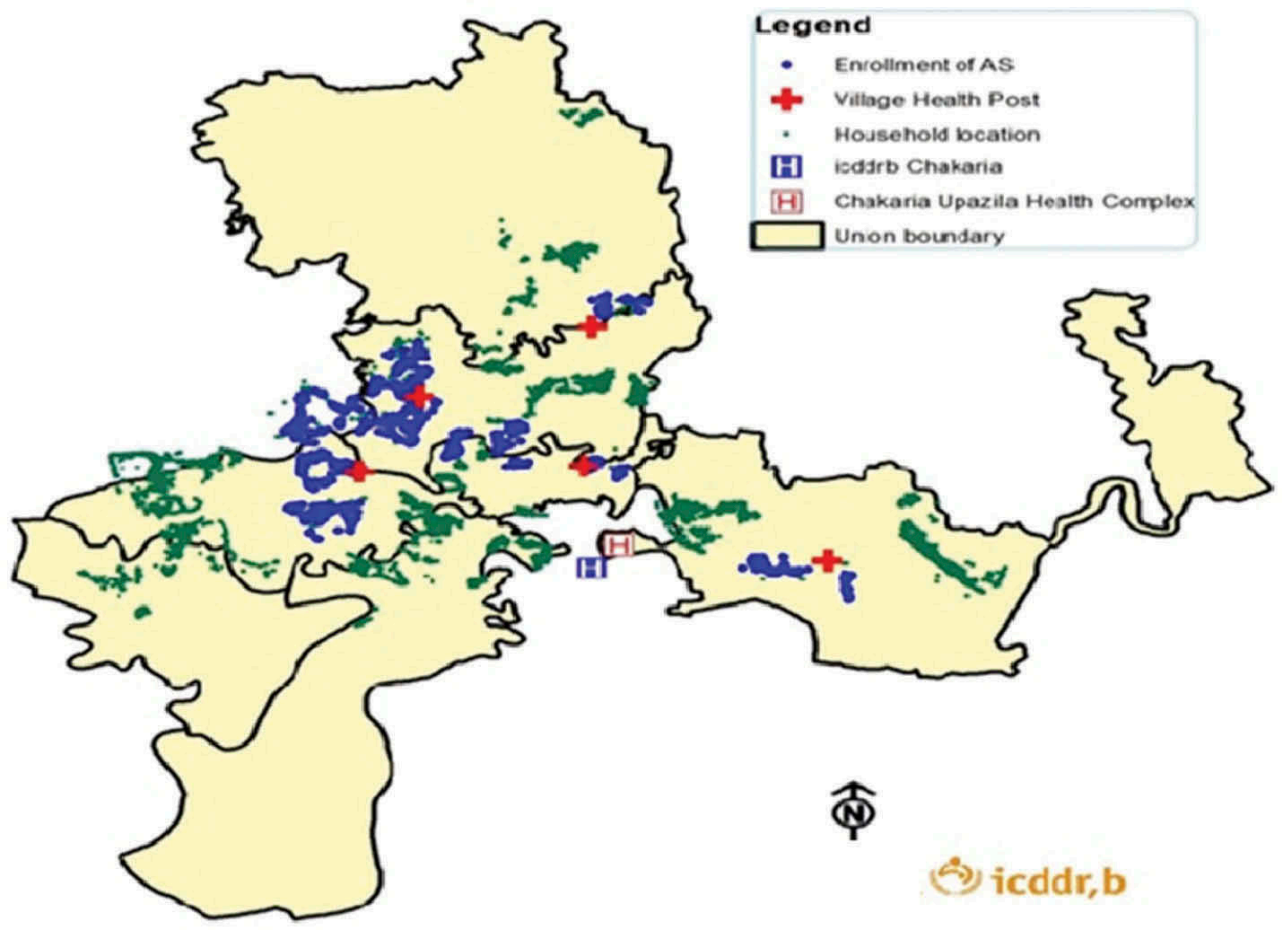
MHI service points and member households in the Chakaria HDSS area.

The MHI catchment area includes 76 villages with 25,513 households and a population of 153,612. Figure 1 shows the Chakaria HDSS area with the locations of VHP and MHI-enrolled households. A household is defined as a unit comprising a single individual or a group of related or unrelated individuals who live in the same compound and share food from the same kitchen. Individuals who live outside the household but spend at least one night every month at the household are also considered as members of the household [12].

### MHI scheme

The AS scheme is a mixture of provider and insurer models. The outpatient services include consultation with paramedics and physicians and are provided through the four VHP established by the community in Chakaria in the late 1990s. The inpatient services for AS members requiring hospitalization on referral from project physicians are provided by Zamzam Hospital, a private facility situated in Chakaria town centre. The outpatients who are members of the MHI scheme can buy their prescribed medicines from partner pharmacies of the scheme, and a part of the cost of drugs is paid by the project to the pharmacy after submission of bills by the pharmacy and verification by relevant personnel of the scheme. The hospital also claims its bills to the project after providing the services. Initially, all payments were paid in cash; however, these are now paid through account-payee cheques from icddr,b.

The scheme operates two benefit packages: (1) inpatient and (2) outpatient. The premium for the inpatient package (termed as the ‘general package’ at the starting phase of the project) is BDT 1200/household/year.

The premium for the outpatient package is BDT 500/household/year. A safety net is built into the outpatient package known as the special outpatient package, and households belonging to the two lower asset quintiles are eligible for this package. The premium for this package is set at BDT 200/household/year. Sometimes, the better-off households in the area donate these special outpatient packages to the poorer households. The premium is collected at the time of enrolment or renewal. It is a one-time payment. At one point of the scheme, paying the premium in instalments was allowed. However, those who were not able to pay the next instalment hesitated to use the health service in times of need. Currently, no payment is accepted in instalments.

The benefits for all the packages include consultation by paramedics and physicians at the four VHP located in a central location in four unions (a civil administrative unit covering a population of 15,000 on average). All members of the scheme are entitled to a 20% discount on medicine costs and discounted hospitalization services in a general ward and other related costs, including medicines, investigation and diagnosis in a private partner hospital. The annual maximum benefit for a household is BDT 54,000 for the inpatient package and BDT 30,000 for the outpatient package and special outpatient package (Table 1).

**Table 1.** Descriptions of packages and benefits of *Amader Shasthya*.

Packages		Benefits
**Outpatient package** Membership or renewal fee: BDT 500 Individual benefit ceiling: BDT 5000 Total household benefit ceiling: BDT 30,000		Treatment from trained Medical Assistant and M.B.B.S. doctors All medicines at 20% discounted price Discount facility on lab examination and treatment at partner hospital
Special outpatient package (subsidized for poor households) Membership or renewal fee: BDT 200 Individual benefit ceiling: BDT 5000 Total household benefit ceiling: BDT 30,000		
**Inpatient package** Membership or renewal fee: BDT 1200 Individual benefit ceiling: BDT 9000 Total household benefit ceiling: BDT 54,000		Treatment from trained Medical Assistant and M.B.B.S. doctors Admission at referral hospital if necessary (during admission at hospital, expenses for general bed, medicines, pathological tests, x-ray, ultrasonography and operation are provided) All medicines at 20% discounted price Discount facility on outdoor service at partner hospital

M.B.B.S.: Bachelor of Medicine and Bachelor of Surgery.

Membership for any of the 3 packages is valid for 12 months, after which it needs to be renewed for continuation of benefits. The members are reminded about renewal by a phone call three months prior to the expiry of membership.

### Data

Data for this paper came from the real-time management information system (MIS) of the scheme and from the HDSS of icddr,b that collects data through quarterly household visits since 1999. Informed written consent is taken from all study participants. The data used in this paper cover the period from the launching of the scheme in February 2012 to May 2015. Programme data that are regularly collected include date of enrolment and renewal of MHI membership, demographic and socioeconomic information of enrolled members, date of utilization of inpatient and outpatient services, diagnosis and drugs prescribed, diagnostic tests advised, amount of claim and disbursement, date of hospitalization with indications and treatment including surgery. Demographic and socioeconomic data on enrolled members from the HDSS area were extracted from the HDSS database using the unique household IDs.

As households enrolled at different time points, the MHI members have not all had the same exposure period and, consequently, the higher the duration of a membership under the MHI the higher the likelihood of receiving a higher amount of benefit for healthcare expenditure and more visits at health facilities. Thus, to keep homogeneity in terms of exposure period among the MHI member households, we have concentrated on the first renewal and its associated determinants. Hence, the dependent variable was a dichotomous variable indicating whether membership was renewed after completion and expiry of the first-year contract. The independent variables included frequency of visits to a facility for receiving health services, amount of benefit received during the preceding one year (in BDT), distance of a member’s residence from the nearest VHP, asset quintile, sex, age, and educational qualification (years of schooling) of the household head.

Regarding proximity of residence to the VHP, the MHI members who reside within 3 km of the nearest VHP were considered ‘adjacent households’ and those who reside further away were said to be ‘non-adjacent households’.

Household economic status was assessed by asset quintiles derived from assets owned, using principal component analysis [17]. The other independent variables were categorized as per the evidence of similar studies [6,7,18] and distribution of programme data.

### Analysis

Cross-tabular analysis was carried out to assess the statistical association between renewal status and independent variables. Stepwise logistic regression analysis was carried out to arrive at a parsimonious model and estimate the net effects of the independent variables on renewal of membership.

Median amount of benefit received for inpatient and/or outpatient care expenditure was determined as the threshold level of benefit regarding healthcare expenditure for membership renewal of the inpatient and outpatient packages.

## Results

**Figure 2.**
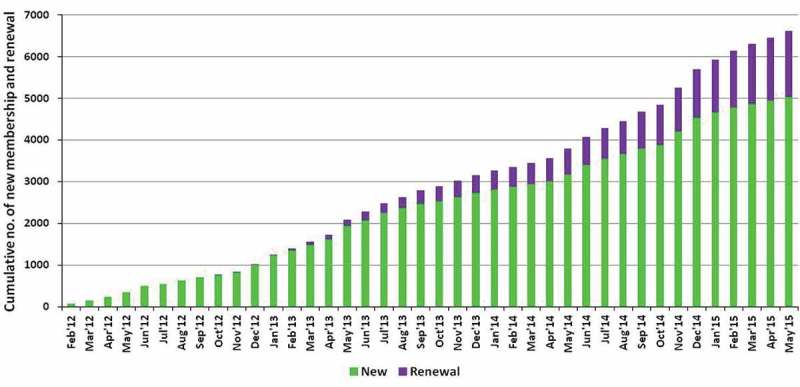
Monthly trend of cumulative numbers of new memberships and renewals of MHI

Figure 2 presents the monthly cumulative enrolment and renewal of membership for the scheme. A total of 5021 households (20%) out of 25,513 households in the catchment area ever enrolled in the scheme during February 2012–May 2015. Both enrolment and renewal showed an increasing trend over time. Of the 5021 enrolled households, 1832 (36.5%) were yet to complete the 12-month membership, implying that they were not yet eligible for renewal. Finally, 3189 households were found eligible for renewal and, of them, 1204 renewed their memberships after completion of the first year. The renewal rate came to be 37.8%. In case of inpatient, outpatient and special outpatient package renewal rates were 44.4%, 26.9% and 42.6%, respectively.

Table 2 presents results of the bivariate analysis showing the associations between renewal of membership and various independent variables. Of the independent variables, frequency of visits to health facilities for receiving health services, amount of benefit received, proximity of member’s residence to the nearest VHP, asset quintile, and educational qualification (years of schooling) of household head showed statistically significant associations with renewal of membership for the inpatient and outpatient packages. For the special outpatient package, no independent variables showed any significant association with membership renewal.

**Table 2.** Socioeconomic and programmatic determinants of membership renewal of MHI, Chakaria, Bangladesh, 2012–15.

	Packages					
	Outpatient package		Inpatient package		Special outpatient package	
Independent variables	N	% renewed	N	% renewed	N	% renewed
**Frequency of visit to VHP/other selected health facilities for receiving health services**						
0 (Used no services)	519	13.3	876	45.2	473	40.2
1–4	365	24.1	250	33.6	156	45.5
5–8	115	49.6	96	37.5	24	54.2
9+	139	66.2	153	61.4	23	60.9
*p*-value	<0.001		<0.001		0.105	
**Proximity of residence to VHP**						
Not adjacent	144	11.8	40	27.5	42	28.6
Adjacent	994	29.1	1335	44.9	634	43.5
*p*-value	0.000		0.029		0.058	
**Asset quintile**						
Lowest	151	17.9	54	38.9	425	41.6
Second	225	21.3	135	24.4	199	47.7
Middle	390	23.8	414	36.0	52	30.8
Fourth	212	38.2	357	47.1	–	–
Highest	160	35.6	415	57.6	–	–
*p*-value	<0.001		<0.001		0.201	
**Age of household head**						
< 30 years	230	26.5	162	40.1	98	30.6
30–44 years	482	27.4	589	46.7	331	45.3
45–54 years	221	27.6	278	44.6	130	43.1
55+ years	203	24.6	346	42.2	117	44.4
*p*-value	0.884		0.371		0.074	
**Educational qualification (years of schooling) of household head**						
0	268	21.3	154	26.0	413	45.8
1–5	426	23.0	497	35.4	232	37.1
6–10	307	32.2	469	50.7	28	39.3
11+	137	38.0	255	61.2	3	66.7
*p*-value	<0.001		<0.001		0.143	
Total	1138	26.9	1375	44.4	676	42.6

### Socioeconomic status, proximity to health centre and renewal

For both inpatient and outpatient packages, socioeconomic status (SES) as measured by the asset index showed a statistically significant influence on decision for renewal. Those belonging to the highest and the fourth quintiles renewed more than those belonging to lower quintiles.

In the case of members for the inpatient, outpatient and special outpatient packages, with heads of the households aged 30 years and above, renewal rates were found to be higher but not significantly so compared to their counterparts with younger household heads (aged below 30 years).

In the case of members for the inpatient and outpatient packages, renewal rates among households where the head had an educational level of Class 6 and higher were found to be significantly higher than among their counterpart households, where the heads had a lower level of education (Class 5 or below). In the case of the special outpatient package, renewal rate did not differ significantly between the abovementioned types of household head.

Considering distance to the nearest health facility, results showed that members residing in villages adjacent to VHP had significantly higher renewal rates than their counterparts in villages non-adjacent to VHP

### Service utilization, benefit claims and renewal of membership

In the case of the inpatient and outpatient packages, renewal of membership was found to be significantly higher among those who availed of services nine or more times compared to their counterparts who availed of services fewer than nine times. For the special outpatient package, the relationship between service utilization and renewal was not statistically significant. In terms of service utilization among members of the inpatient package, renewal was highest (61.4%) among those who used services at least 9 times during the 1 year preceding renewal. However, the renewal rate was found to be around 45% (higher than those who used services between 1 and 8 times) for those who did not use any services.

In the case of members of the inpatient and outpatient packages, membership renewal among those who received the median amounts of BDT 1205 and BDT 502 or more respectively as benefit, was significantly (*p* < 0.001) higher than among their counterparts who received less than those amounts or no benefit at all (Table 3).

**Table 3.** Membership renewal of MHI by amount of benefit received from MHI, Chakaria, Bangladesh, 2012–15.

Independent variables	Inpatient package	
	N	% renewed
**Amount of benefit received against healthcare expenditure (BDT)**		
0 (Used no services)	876	45.2
< 1205	237	24.9
≥ 1205	262	59.2
Total	1375	44.4
*p*-value	0.000	
	**Outpatient package**	
	**N**	**% renewed**
**Amount of benefit received against healthcare expenditure (BDT)**		
0 (Used no services)	519	13.3
< 502	291	22.7
≥ 502	328	52.1
Total	1138	26.9
*p*-value	0.000	

### Multivariate analysis

Tables 4 and 5 present the results of stepwise logistic regression analysis for the fitted model with different independent variables to explore their relative association with renewal of membership for the inpatient and outpatient packages. As none of the independent variables showed any significant influence on renewal of membership for the special outpatient package in the bivariate analysis, the logistic regression analysis was not carried out for renewal of this particular package.

**Table 4.** Odds ratio of factors associated with renewal of membership of outpatient package of MHI scheme of Chakaria (Model 1 and Model 2).

Independent variables	Model 1	Independent variables	Model 2
**Frequency of visit to VHP/other selected health facilities for receiving health services**	***	**Amount of benefit received against healthcare expenditure (BDT)**	***
0 (Ref)	1.0	0 (Ref)	1.0
1–4	2.1***	< 502	1.8**
5–8	6.4***	≥ 502	7.2***
9+	14.1***		
**Proximity of residence to the VHP**	***	**Proximity of residence to the VHP**	***
Not adjacent (Ref)	1.0	Not adjacent (Ref)	1.0
Adjacent	2.9***	Adjacent	3.3***
**Asset quintile**	*	**Asset quintile**	***
Lowest (Ref)	1.0	Lowest (Ref)	1.0
Second	1.0	Second	1.0
Middle	1.0	Middle	1.1
Fourth	1.7	Fourth	2.2**
Highest	2.0*	Highest	2.7**
**Educational qualification (years of schooling) of household head**	*		
0 (Ref)	1.0		
1–5	0.9		
6–10	1.5		
11+	1.9*		

Notes: Ref = Reference category; *Significant at *p* < 0.05, **p < 0.01, ***p < 0.001; Educational qualification (years of schooling) of household head variable was eliminated from Model 2 in the last steps of logistic regression analysis.

**Table 5.** Odds ratio of factors associated with renewal of membership of inpatient package of MHI scheme of Chakaria (Model 1 and Model 2).

Independent variables	Model 1	Independent variables	Model 2
**Frequency of visit to VHP/other selected health facilities for receiving health services**	***	**Amount of benefit received against healthcare expenditure (BDT)**	***
0 (Ref)	1.0	0 (Ref)	1.0
1–4	0.8	< 1205	0.5***
5–8	0.8	≥ 1205	2.2***
9+	2.4***		
**Proximity of residence to the VHP**	*	**Proximity of residence to the VHP**	**
Not adjacent (Ref)	1.0	Not adjacent (Ref)	1.0
Adjacent	2.7*	Adjacent	3.0**
**Asset quintile**	***	**Asset quintile**	***
Lowest (Ref)	1.0	Lowest (Ref)	1.0
Second	0.4*	Second	0.5*
Middle	0.6	Middle	0.7
Fourth	0.8	Fourth	0.9
Highest	1.1	Highest	1.3
**Educational qualification (years of schooling) of household head**	***	**Educational qualification (years of schooling) of household head**	***
0 (Ref)	1.0	0 (Ref)	1.0
1–5	1.4	1–5	1.4
6–10	2.3***	6–10	2.2***
11+	3.3***	11+	3.1***

Notes: Ref = Reference category; *Significant at *p* < 0.05, **p < 0.01, ***p < 0.001.

The correlation between amount of benefits received and frequency of service utilization was high (0.5, *p* < 0.01) and, to avoid multicollinearity, we fitted separate models for these two variables. The analysis that uses ‘frequency of service utilization’ as one of its independent variables is labelled as Model 1 and the one that uses ‘benefit received’ instead of ‘frequency of service utilization’ as one of its independent variables is labelled as Model 2 hereafter.

#### Model 1

Tables 4 and 5 present the results of stepwise logistic regression analysis for the fitted Model 1 with renewal of membership as dependent variable and the following as independent variables: frequency of visit to facilities, proximity of residence to the VHP, asset quintile, age of the household head and years of schooling of household head.

In the case of both inpatient and outpatient packages, four independent variables (frequency of service utilization, proximity of residence to the VHP, asset quintile, and years of schooling of household head) revealed significant associations with renewal of membership.

For the inpatient and outpatient packages, odds of renewal of membership were 2.4 and 14.1 respectively for those who visited 9 or more times compared to those who did not visit health facilities at all. In the case of members for the inpatient package, members who received services 1–4 or 5–8 times were found to be less likely to renew membership than members who visited 9 or more times.

In the case of members for the inpatient and outpatient packages, members whose residences were within 3 km of a VHP were 2.7 and 2.9 times respectively more likely to renew membership than their counterparts whose residences were beyond 3 km from the nearest VHP Membership renewal for the outpatient package was found to be 1.7 and 2.0 times more likely among those belonging to the fourth and the highest asset quintile respectively compared to that of the members belonging to the lowest quintile. For the outpatient package, households with educated household heads were more likely to renew than those with illiterate household heads. The odds of renewal were 1.5 and 1.9 for households with the household head’s schooling being 6–10 years and 11+ years, respectively, compared to those with illiterate household heads.

In the case of the inpatient package, members in the highest asset quintile were equally likely to renew membership compared to those in the lowest quintile. Household heads of members of the inpatient package, with 6–10 and 11+ years of schooling, were found to be 2.3 and 3.3 times more likely to renew membership compared to those members whose household heads were illiterate.

#### Model 2

To explore the relative association of amount of benefit received by members for the inpatient and outpatient packages with renewal of membership, two stepwise logistic regression models were fitted with independent variables: amount of benefit received, proximity of residence to VHP, asset quintile, and age and years of schooling of the household head.

In Table 5, results of stepwise logistic regression analysis revealed that all independent variables, except age of household head, had significant associations with renewal of membership for the inpatient package. Members for the inpatient package, who received benefit amounting to BDT 1205 or more, were found to be 2.2 times more likely to renew membership compared to their counterparts receiving no benefit when the effects of other variables were controlled for. Members of the inpatient package from the highest quintile were found to be 1.3 times more likely to renew membership compared to those from the lowest quintile after controlling for amount of benefit received and other independent variables. Members of the inpatient package with household heads having 6–10 years and 11 or more years of schooling were 2.2 and 3.2 times more likely to renew membership respectively, compared to those whose household heads had no education.

In Table 4, the relative associations of independent variables with membership renewal for the outpatient package are presented, which indicate that outpatient package members receiving BDT 502 or more were 7.2 times significantly more likely to renew membership than their counterparts who received no benefit for healthcare expenditure. The outpatient package members in the fourth and the highest asset quintiles were respectively 2.2 and 2.7 times significantly more likely to renew membership compared to their counterparts in the lowest quintile while the effect of amount of benefit received, and other independent variables, were controlled for. The outpatient package members whose households are within 3 km of a VHP were found to be 3.3 times more likely to renew membership compared to those members whose households are beyond 3 km from the nearest VHP

In the case of membership renewal for the outpatient package, the associations of independent variables, such as proximity of residence to VHP, asset quintile, and educational qualification (years of schooling) of household head, differ to some extent when controlled separately for frequency of service utilization and the amount of benefit received for healthcare expenditure, along with other independent variables. In the case of membership renewal for the inpatient package, the odds of associated factors of the two models, Model 1 and Model 2 (as mentioned previously), differed very slightly.

## Discussion

Some of the most important questions related to voluntary MHI, like the scheme presented in this paper, include: Does MHI have any potential in settings like Bangladesh? Will people enrol and continue membership in such schemes? What implications do the lessons learned have for health insurance in general? Fundamental to these questions are the enrolment and renewal prospects of MHI schemes. Findings from the current study are, thus, crucial as it identifies socioeconomic and programmatic determinants of membership renewal which can help programme managers to modify programmatic approaches to increasing enrolment and renewal. The study is based on high-quality programmatic data generated from the real-time online MIS of the scheme and socioeconomic data from the regular HDSS run by icddr,b.

The cumulative enrolment rate over the first 3 years of operation of the MHI scheme studied in this paper was reported to be 20%, and the first-time renewal rate was 38%. Generally speaking, these rates may seem low but, in fact, they are better than or at least as good as those that have been observed so far in other similar settings. The enrolment rates in other settings have been in the range of 15–20% [7], and the renewal rates varied from 7% in Nicaragua [8], to 4% in India [19], with exceptions of 54% and 65% in Burkina Faso and Nepal, respectively [9,10]. The renewal rate of 38% found in the present study is of particular interest, which indicates that people in a community unfamiliar with insurance and risk-pooling saw benefits of enrolling into an MHI scheme and, hence, also renewed membership. However, challenges remain in increasing the rate of renewal further.

The study found that the most important determinants of renewal were frequency of consultation and amount of benefit received by clients. The relationship was found to be more explicit for members of the outpatient package compared to the inpatient one. For members of the outpatient package, likelihood of renewal increased with an increase in consultation and benefit amount whereas for members of the inpatient package the results were mixed. Renewal rate was comparatively high among those who did not use services at all compared to those who used services 1–8 times. A possible explanation could be that households that enrolled into the inpatient package were those who could afford the premium (which is more than twice the outpatient package) and were more inclined to avoid the risk of catastrophic health expenses. Perhaps these are the group of people who were more aware of and convinced about the benefits of health insurance. We also found that the renewal rate, in general, was high among members of the inpatient package compared to members of the outpatient package (44.4% vs 26.9%). Thus for the renewal of inpatient membership no significant difference was found between two broad categories, i.e. those who utilized services 1–8 times and those who did not.

A threshold of visit frequency and benefit received was, however, identified from the findings. Results showed that nine or more visits increased the likelihood of membership renewal substantially for both outpatient and inpatient packages (the likelihood of renewal for the inpatient package was one-sixth of that for the outpatient package) compared to those making fewer visits. For the amount of benefit received, a threshold of BDT 1205 or more for the inpatient and BDT 502 or more for the outpatient package was found to encourage renewal. A likely explanation of this phenomenon could be that the increased consultation with care providers tends to earn the trust of their clients when people get what they were promised for; this trust convinces them to continue investing in such schemes. Trust has played a major role in enrolment and renewal of membership in similar schemes in other settings as well [11,20].

Further, a positive relationship was found between education of the head of household and renewal, which is an expected outcome because it is likely that the educated members are open to innovative ideas, more aware about the unknown nature of health crises and their financial consequences, which increases their preference for risk aversion [21], and are more convinced about the benefits of such a scheme than the others [11].

Proximity of a member’s residence to a service centre providing especially outpatient services was found to be associated with higher probability of renewal. Though association persists between proximity and service use variables, these were not eliminated from the model until the last steps of logistic regression analysis and both variables were found to be significant covariates for membership renewal. Availability of healthcare services from places closer to the residence of patients is less time-consuming and also requires lower transport costs. Transport costs and travel-time have been found to have significant effects on enrolment and renewal in MHI schemes in earlier studies [11,22]. Thus, it is likely that any scheme of this kind will benefit from its presence with healthcare services being close to the community it serves, to ensure higher enrolment and renewal.

For members of the inpatient package, households belonging to the highest asset quintile were more likely to renew membership even after controlling for the amount of benefit received by the households. This finding is of great concern as households belonging to the lowest SES are usually in greater need of protection against higher OOP expenses compared to the better-off households that have a comparatively higher ability to pay [23]. Safety nets which provide services at a subsidized rate for the households with lower ability to pay could play a positive role in this aspect and can go a long way in ensuring UHC in the country.

Given the recent initiative of the government of Bangladesh to establish a social health insurance mechanism as a step towards achieving UHC to meet the targets of the SDG [24], the findings presented in this paper can be of great importance. The current health financing strategy (2012–2032) of the country has emphasized the role of small-scale MHI or community-based health insurance schemes in the interim period before the country launches its social health insurance scheme [25]. Further, as this paper highlights, challenges remain in terms of enrolment in and renewal of MHI schemes. However, a well-designed MHI scheme can play a complementary role to the national system.

Many communities in Bangladesh continue to experience high unmet need in relation to free services provided in public-sector health facilities as a result of shortage of supply, a high patient–doctor ratio, long waiting times, unofficial payments, and biased treatment for people of varying SES [5,26]. MHI can work towards bridging the gap between what people need and what the public-sector healthcare facilities offer, particularly in hard-to-reach areas. Depending on their suitability, it is also possible to use MHI as insurance providers while healthcare provision is made through the public- and private-sector providers, i.e. a purchaser–provider split model. This model has the potential to induce healthy competition in the market and thereby improve quality of services and efficiency in the overall health systems of the country. Having said this, it is also recognized that schemes such as MHI or community-based health insurance (CBHI) cannot possibly cover the whole population of the country and be the sole contributor in achieving UHC Complementary financing mechanisms will also be needed. The approach being tested in Bangladesh where MHI or CBHI will lead to a social health insurance scheme and the premium for the poor will be paid by the government, is similar to that of Indonesia, which resulted in coverage of 76.4 million poor people [27]. In Viet Nam, the Health Care for the Poor Program (HCFP) offered a substantial improvement in access to healthcare among the poor through an increase in healthcare utilization [28,29] and a reduction in healthcare spending [29,30]. Other potential effects of the MHI programme include the enhanced use of preventive care and reduction of adverse financial consequences due to illnesses.

## Limitations of the study

Two important limitations of the study are worth mentioning. First, as the study used only programmatic data, it was not able to address some independent variables, including users’ and care providers’ perspectives on membership renewal, copayment, cost of alternate health service etc. Second, the scheme analysed in this paper was run by a very reputable and trusted organization in Bangladesh (icddr,b), which contributed to the discovery of oral rehydration solution and also runs cholera hospitals [11,20,31,32]. In matters of MHI, trust in the implementing organization is important and, thus, the findings might have been influenced by the reputation of icddr,b which could not be disentangled from the other variables, and perhaps limits the generalizability of the findings. However, it does not necessarily overestimate the association between the determinants and membership renewal for MHI

## Conclusion

It can safely be said that enrolment as well as renewal are crucial for initiation and continuation of MHI, which can prepare the ground for designing and implementing a social health insurance programme. In addition, MHI has the potential to provide learning for other modes of health protection schemes and provide an opportunity to study human behaviour in response to risk-pooling and prepayment, which has relevance for other programmes of a similar or near-similar nature. The concept of health insurance being relatively new in settings like Bangladesh, there is a clear need for continuous learning on the schemes with far-reaching implications for financial protection mechanisms to achieve UHC

## Paper context

Micro health insurance (MHI) is a tool to reduce out-of-pocket healthcare costs. Low renewal rates of MHI are observed generally. Inadequate understanding about the determinants of renewal exists mostly because of lack of required data. This paper analyses programmatic and community-based data from a rural area of Bangladesh with a MHI in place since 2012. Amount of benefit, frequency of use of the scheme and proximity to service points were important factors associated with renewal.
